# Identification and Characterization of the Antigens Expressed On the Surface of Human Erythrocytes Infected With *Plasmodium falciparum*

**Published:** 2013

**Authors:** N Kalantari, S Ghaffari

**Affiliations:** 1 Cellular and Molecular Biology Research Center, Babol University of Medical Sciences; Laboratory Sciences Group, Faculty of Paramedical, Babol University of Medical Sciences, Babol, Iran; 2 Parasitology and Mycology Group, Faculty of Medicine, Babol University of Medical Sciences, Babol, Iran

**Keywords:** Cytoadherence, *Plasmodium falciparum*, *Pf*EMP1, Surface antigens

## Abstract

**Background:**

Molecules expressed on the surface of infected erythrocytes (IE) with *Plasmodium falciparum* play important roles in malaria pathogenesis and immune evasion. Some of these molecules are specific adhesive ligands mediating adhesion of IE to the vascular endothelium. In the current study, the antigens exposed on the surface of IE with different isolates and various binding subpopulations of *P. falciparum* were studied.

**Methods:**

A pooled hyper immune serum (HIS) from Malawian adults and eluted antibodies from the surface of the homologous and heterologous parasites were used. The parasite surface molecules were analyzed by Immuno-Gold-Silver enhancement (IGSE) and Western blotting. Mini-column cytoadherence method was used to select various parasite-binding subpopulations.

**Results:**

Surface antigens of all the isolates were recognized by HIS and high recognition of antigens was observed in all isolates with homologous eluted antibodies. Western blot analysis showed that the eluted antibodies reacted with a small subset of antigens compared with HIS. Three bands, *Pf*EMP-1, were detected in the Triton X- insoluble fraction of the ICAM-1 binding subpopulation. Another interesting band was ∼ 52-55 kDa in various isolates of *P. falciparum*. This molecule as defined by its low molecular weight, Triton X-100 solubility, surface location and sensitivity to 1 mg/ml trypsin.

**Conclusion:**

The IE's surface antigens differed in parental population compared with the selected subpopulations. These molecules could induce isolate-specific immunity. Antibodies purified from the surface of IE can be used as specific reagents to investigate parasite-derived proteins expressed on the surface of IE.

## Introduction

*Plasmodium falciparum* causes the most severe form of malaria in humans with about two million deaths annually (1). The disease symptoms are completely associated with the erythrocytic phase of infection where parasite multiplication takes place. As the parasite develops in the erythrocytes, several changes such as modifications of the cell membrane, changes in metabolite transport and the insertion of a number of parasite-derived proteins into the surface of the infected erythrocyte membrane, occur (2). The molecules exposed on the surface of infected erythrocytes, particularly, *P. falciparum* erythrocyte membrane protein-1 (*Pf*EMP-1) and rifins, play important roles in the pathology of severe disease, anti-parasite immunity and cytoadherence to the endothelial receptors (3–4). The best known protein on the surface of infected erythrocytes is *Pf*EMP-1. This protein is strain-specific and its biochemical characteristics are insolubility in non-ionic detergents, sensitivity to proteases, high molecular weight and high polymorphism (5). These proteins have been identified as the *var* family, which is encoded by 60 *var* genes (6). Switching expression between different *var* genes allows the parasite to evade the host immunity and may change disease manifestations by modifying infected erythrocytes adhesion trait (3, 7). Therefore, each parasite population represents a mixture of different subpopulations with different binding characteristics (8–9).

Moreover, several studies have found a correlation between specific parasite adhesion character and disease outcome (10–12). Among various host receptors, adhesion to CD36 and intercellular adhesion molecule 1 (ICAM-1) are the most common adhesion trait in the parasite populations (11, 13–14) and can synergize under flow conditions to mediate infected erythrocyte bind to microvasculature endothelium (15). Some studies demonstrated that adhesion of infected erythrocytes with *P. falciparum* to ICAM-1 has associated with cerebral malaria (11), but this association was not seen by others (13–14, 16). In addition, rosetting, binding of the infected red blood cells to uninfected red blood cells, has been associated with disease severity in African children (17–19). On the other hand, immunity to falciparum malaria is incomplete and is associated with parasite derived red cell surface antigens, particularly the *Pf*EMP1 proteins (20–21). Some studies indicate that immunity to *P. falciparum* is strain specific (22–'^23^) and others studies demonstrate cross-reactive antibodies to surface antigens of different *P. falciparum* isolates (24–26).

In this paper, we report the application of mini-column cytoadherence method to select parasite-binding subpopulations and application of purified antibodies from the surface of infected erythrocytes as a specific reagent. These performed to identify expressed proteins on the surface of infected red blood cells and contribution of them in cytoadherence.

## Materials and Methods

### Parasites and cells

Three *P. falciparum* linesA4 (7, 15), 3D7 (from NF54 from Netherland received from D. Walliker), Indochina-1(CDC, adapted to Saimiri monkey) and two Malawian isolates were used. All parasites were cultured in human blood group O ^+^ using RPMI-1640 containing AB^+^ human serum (RPMI-HS) mostly described by Mphande et al.,2008 (27). Chinese Hamster Ovary cells (CHO) or CHO transfected with CD36 or with ICAM-1 cells were cultivated as described by Vogt, 2008 (28). These cells were kindly prepared by Dr. Russell Howard.

### Sera

A pooled hyper-immune serum from African adults (HIS) (Red Cross Foundation, Central Laboratory, Switzerland), antibodies purified from *P. falciparum* -infected erythrocytes (different clones and various binding subpopulations of A4 lines), antibodies purified from non-infected erythrocytes and a pooled normal human serum from European people were used.

### Elution antibodies preparation

Antibodies were purified from the surface of infected and non-infected erythrocytes using a modified version of method described by Rekvige and Hannestad (29). Briefly, late trophozoites/schizonts of *P. falciparum*-infected erythrocytes were purified with Plasmagel method (30). The cells were washed three times in 2% PBS-BSA and then one volume of the packed cells was mixed with two volumes of 1:2 dilutions of the pooled hyper-immune serum. The cells were incubated for 1 hour at 37°C. During incubation time, the cells re-suspended every 15 minutes by gentle agitation and then centrifuged. The supernatant discarded and the cells were washed three times as before. Two volumes of icy elution buffer (50 mM glycine- HCl buffer, containing 150 mM NaCl, pH = 3) was added to the cells in order to elute antibodies from the surface of infected cells. After 2 minutes agitation the tube was centrifuged and the supernatant collected. The supernatant was immediately neutralized with 1M Tris (Trizma base, Sigma) and stored at -20 °C.

### Cytoadherence on mini-column

Mini-column adhesion assay and selection of a particular binding subpopulation of *P. falciparum* were performed as previously described (9). Briefly, a column was made by suspending Cytodex beads, previously covered with CHO cells, in a 1 ml pipette tip fitted with a polyethylene disc to retain the beads in the column. The column was then washed once with RPMI-1640 containing fetal calf serum (RPMI-FCS) followed by the addition of 1 ml of *P. falciparum* culture at 2% hematocrit. The column was washed three times with RPMI-HS to remove unbound infected erythrocytes. The bound cells were eluted from the column by transferring Cytodex beads to a clean tube and shaking them gently to suspend the cells in the RPMI-HS. After the beads settled, the supernatant was collected, centrifuged and the pellet used for SDS-PAGE and Western blotting.

### Preparation of eluted antibodies from selected- parasite surface antigens using on-column cytoadherence assay

A column was made with CHO/ICAM-1 cells as described before. The column was washed three times and then 5 ml of the HIS (1:2 dilution) was passed through the column three times. After four times washing, 10 ml of the elution buffer (50 mM glycine- HCl buffer, containing 150mM NaCl, pH = 3) was passed through the column and collected the solution off from the column. The low pH of the eluted antibodies was immediately neutralized with 1M Tris (Trizma base, Sigma). Fetal calf serum was added to the eluted antibodies and then stored at -20°C.

### Immunogold-silver enhancement stain (IGSE) and Surface immunofluorescence

The techniques used fresh, unfixed and washed infected erythrocyte as described by Hommel et al., 1991; 1983, respectively (31–32). The assays were performed in two independent experiments and in duplicates.

### Sodium dodecyl sulfate-polyacrylamide gel electrophoresis and Western blotting

The enriched infected erythrocytes with late stages of the parasite were extracted with 1% TritonX-100/PBS containing a cocktail of proteinase inhibitors and subsequently, non-soluble fraction in Triton X-100 was extracted with 2% SDS. The Triton X-100 soluble and SDS soluble fractions were mixed with equal volume of SDS-PAGE sample buffer, boiled for 5 minutes, and separated on 5% polyacrylamide gels. For Western blot analysis, proteins were transferred to nitrocellulose membranes by semidry electrophoresis in Tris-glycine buffer, pH 8.3. Membranes were blocked with PBS containing nonfat dry milk, after which the membranes were incubated for 1 hour in primary antibody solution. The membranes were washed, followed by incubation for 1 hour with secondary antibodies (anti human polyvalent Ig conjugated to horseradish peroxidase) (Sigma). Membranes were developed with Diaminobenzedine and H_2_O_2_ and documented with an imaging system.

### Trypsin treatment of CHO/ICAM-1 binding subpopulation of A4-IRBC

Three columns of CHO/ICAM-1 cells were prepared and cultivated parasite, A4 line, was passed through the columns. The bound cells were collected as explained before and washed once. The washed cells were treated with 1, 0.1, 0.01 mg/ml trypsin (Sigma) for 5 minutes. The cells were washed once and incubated with 2 mg/ml soybean trypsin inhibitor (Sigma) for 5 minutes (33). After two more washings, the cells were stored at -20 °C.

## Results

### Immunostaining experiment

**Fig. 1:**
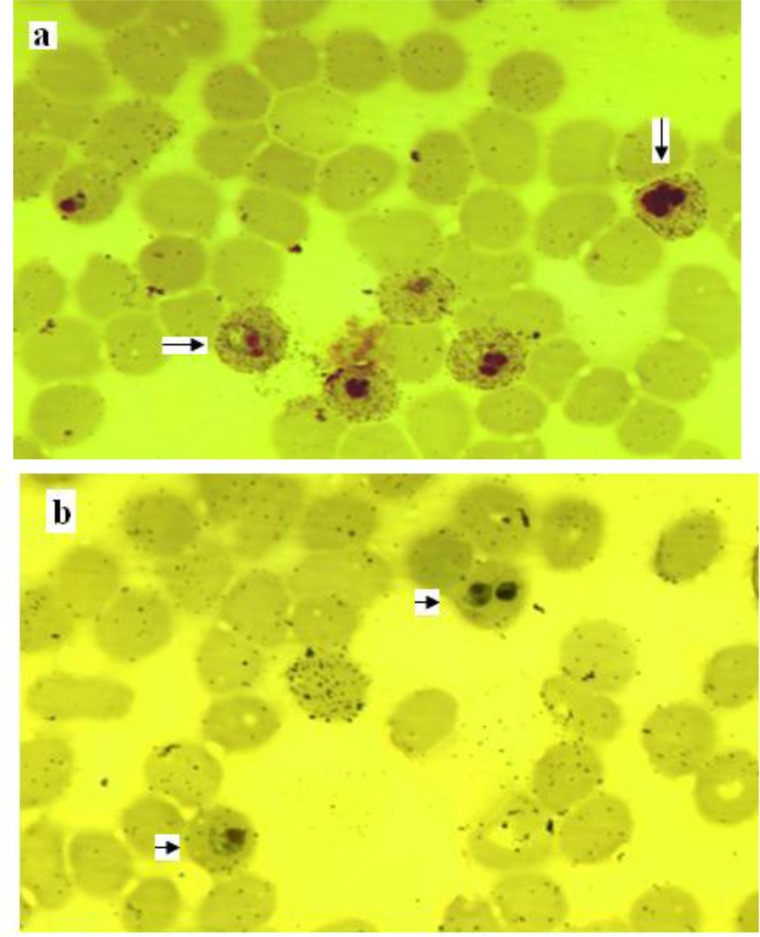
Characteristic results obtained after testing infected cells with an immune serum using a) hyper-immune pool or antibodies purified from infected erythrocytes and b) a negative control using elution antibody from normal red blood cells or from heterologous isolates. Arrows denote positive and negative staining. The 1000 X magnification with oil was used

IGSE and surface immunofluorescence were carried out for three different clones of *P. falciparum* (A4, Indochina and 3D7) using HIS and eluted antibodies form the surface of homologous, heterologous isolates and uninfected red blood cell. The results showed that antigens expressed on the surface of infected erythrocytes with all three parasite lines were recognized by the HIS while the eluted antibodies from the surface of a particular parasite line were able to recognize antigens on the surface of homologous parasite. The values obtained for IGSE stained parasites with eluted antibodies from the surface of homologous parasite showed good correlation to the results obtained by the HIS. Few surface antigens were recognized by antibodies eluted from heterologous parasites and antibodies eluted from uninfected erythrocytes (Fig. 1 and Table 1).

**Table 1:** Recognition antigens expressed on the surface of infected erythrocytes with different lines of *P. falciparum* and specificity of eluted antibodies to these molecules using HIS, homologous and heterologous eluted antibodies. The numbers indicate the percentage of infected erythrocytes stained with IGSE which represents results obtained from two experiments and in duplicates. E Ab.A4, E Ab.3D7 and E Ab. Indochina are eluted antibodies from the surface of infected erythrocytes with A4, 3D7 and Indochina clones, respectively. N-RBC is uninfected red blood cell

Sera	HIS	E Ab. N-RBC	E Ab. A4.	E Ab. 3D7	EAb. Indochina IR
Isolates					
A4	95	0	85	2	1
3D7	98	8	7	93	9
Indochina	93	1	2	3	91

### SDS-PAGE and Western blotting

**Fig. 2:**
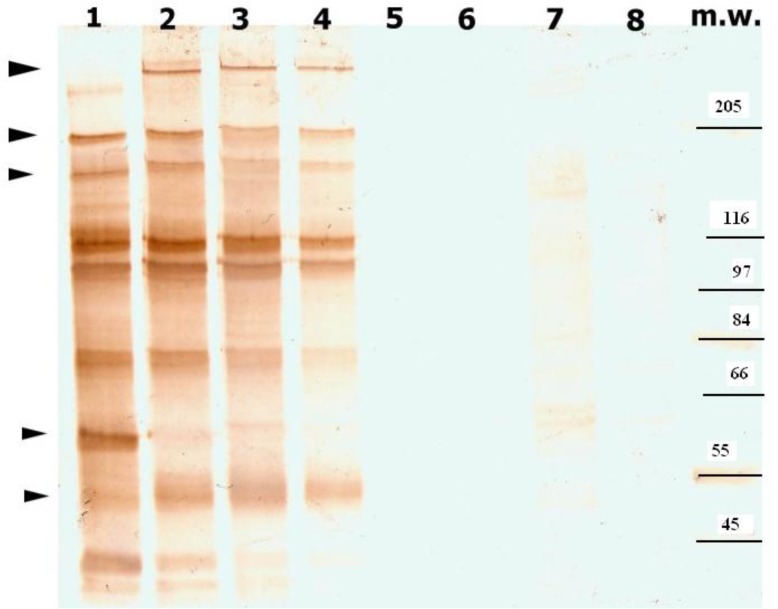
Immunoblot analysis of TS fraction of parental population, ICAM-1/ CD36/CHO-selected subpopulations of A4, uninfected erythrocytes, CHO/ICAM-1, CHO-CD36 and CHO-cells. The proteins extract were loaded on 5% SDS-PAGE and blots were probed by 1:100 dilutions of the HIS. Arrows denote divers molecules in parasite extract/ Lines are: 1. parental population of A4, 2.ICAM-1 binding subpopulation, 3.CD36 binding subpopulation, 4.CHO binding subpopulation, 5.uninfected erythrocytes, 6.CHO/ICAM-1 cells, 7.CHO/CD36 cells, 8. CHO cells

SDS-PAGE and Western blot analysis showed that various parasite lines and different binding subpopulations of A4 clone of *P. falciparum* generated different pattern of bands. For example, a band was detected in the TS fraction of various binding subpopulations of A4 which was divers and had high molecular weight (approximately 264 kDa). This molecule was not seen in the TS fraction of unselected parasite or uninfected erythrocytes and CHO cells but we found a ∼246 kDa molecule in parental population of A4 (Fig. 2).

In addition, another diverse molecule was detected which had molecular weight ranging from ∼198 kDa to 205 kDa. Other divers molecules which had ∼60 kDa and ∼55 kDa molecular weight were seen in the extracts of different binding subpopulations of A4 isolate (Fig. 2). Immunoblotting of proteins extracts of the ICAM-1 binding subpopulation of two field isolates, ICAM-1 binding subpopulation of A4 and parental population of A4 demonstrated that a high molecular weight protein was detected in all parasite population extracts When the blot probed with the HIS. The molecular weight of these proteins were ∼ 246 kDa, ∼ 263 kDa, ∼ 249 kDa and ∼ 262 kDa in the TS extracts of A4, ICAM-1-selected subpopulation of A4, ICAM-1 binding subpopulation isolates number 350 and 272, respectively. When the blots were probed by the antibodies eluted from the surface of ICAM-1 binding subpopulation of A4, only ∼246 kDa and ∼264kDa molecules were identified in the parental and ICAM-1 binding subpopulation of A4 (data are not shown). These finding revealed that purified antibodies from the surface of infected erythrocytes with a particular isolate are able to recognize antigens expressed on the surface of homologous line. Also, a low molecular weight protein, ∼ 55 kDa, ∼52-53 kDa, in the ICAM-1 binding subpopulations of A4 and field isolates was detected by the HIS and the eluted antibodies. Another band was seen in the ICAM-1 selected subpopulation of A4 (∼ 42kDa) but not in the ICAM-1 binding subpopulations of the field isolates. Furthermore, other diverse molecules were seen at ∼ 205, ∼116, ∼84 kDa which were recognized by both HIS and the eluted antibodies.

In order to further characterization of the high and low molecular weight proteins in the parasite extract, ICAM-1 binding subpopulation of A4 was obtained by mini-column cytoadherence technique. The retained infected cells were collected and cultured. The mature stage of the fourth generation of the selected parasites was divided to 2 parts, one was used to repeat the adhesion assay and another part was purified by Plasmagel to prepare protein extracts of the fourth generation of the ICAM-1 binding subpopulations. Western blot analysis of the TS fraction of these protein extracts showed that the ∼ 263 kDa was detected in the first and second selected subpopulations of A4 by both immune reagents (HIS and the eluted antibodies) (data are not shown). But, ∼ 245 kDa molecule was seen in the TS fraction of the fourth generation of the selected parasite. In the TI fraction, different bands with high molecular weight were seen in the selected parasite.

### Trypsin treatment of CHO/ICAM-1 binding subpopulation of A4-IRBC

**Fig. 3:**
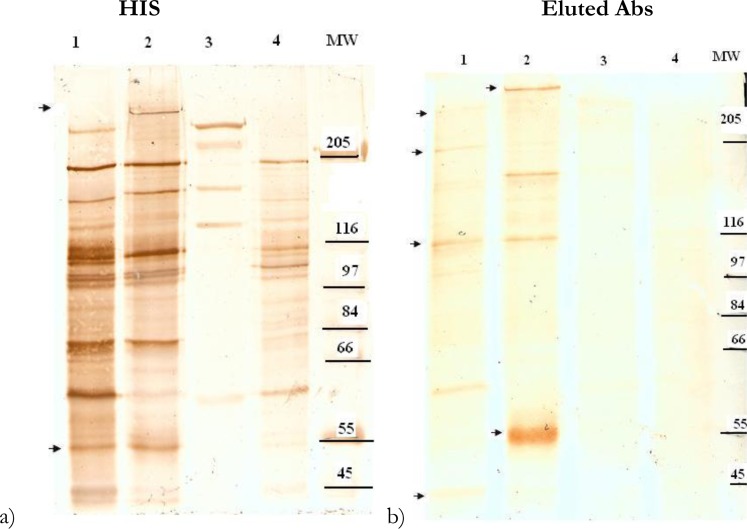
Immunoblot analysis of TS fraction of *P*. *falciparum*- infected erythrocytes and uninfected cells. The samples were loaded on 5% SDS-PAGE and the blots were probed by: a) 1:100 dilutions of the HIS; b) 1:2 dilutions of the eluted antibodies from the infected cells bound to ICAM-1, using on-column technique Lines are:1. Parental population of A4, 2. ICAM-1 binding subpopulation, 3. Uninfected red blood cells, 4. ICAM-1 binding subpopulation of A4 treated with 1 mg/ml trypsin

Controlled proteolytic digestion of intact ICAM-1 binding subpopulation was used to further explore possible associations between the expression of surface antigens and binding phenotypes using HIS and antibodies eluted from ICAM-1 binding subpopulation of A4 parasite. Upon incubation of selected parasite with trypsin, complete deletion of the ∼ 263 kDa and 55 kDa bands in the TS fraction of the selected parasite at 1 mg/ml or higher concentrations of the protease, occurred (Fig. 3).

Furthermore, analysis of the TI fraction of the ICAM-1 binding subpopulation and parental population of A4 showed that various high molecular weight proteins exist in this fraction and that the location of these molecules is different in the selected and unselected parasites.

**Fig. 4:**
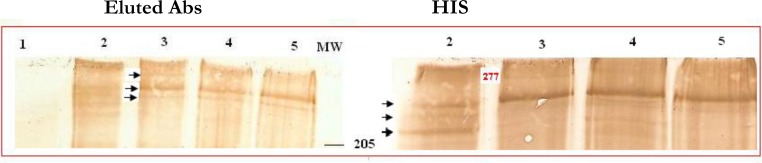
Immunoblot analysis of TI fraction of infected and uninfected red blood erythrocytes. The samples were loaded on 5% SDS-PAGE and the blots were probed by HIS and eluted antibodies from the surface of ICAM-1 binding subpopulation: Arrows denote several high molecular weight proteins detected by both immune reagents and removing 277 kDa proteins by trypsin Lines are: 1. Uninfected erythrocytes, 2. Parental population of A4, 3. ICAM-1 binding subpopulation, 4. Trypsin- treated ICAM-1 binding subpopulation (0.01 mg/ml), 5. Trypsin- treated of ICAM-1 binding subpopulation (0.1 mg/ml).

These bands range from ∼253 to ∼277 kDa in selected parasites and ∼230-262 kDa in unselected parasites. However, complete deletion of the ∼ 277 kDa occurred at 0.01mg/ml or higher amounts of trypsin (Fig. 4). Moreover, this experiment indicated that eluted antibodies from the surface of infected cells bound to ICAM-1 are able to recognize a few molecules in protein extracts of the untreated and treated intact cells with 0.1 and 0.01 mg/ml of trypsin. These bands were completely removed by 1 mg/ml concentration of the enzyme (Fig. 3).

## Discussion

In this work, we demonstrate that antibodies purified from the surface of infected erythrocytes with a particular isolate of *P. falciparum* not only reacted with unique antigens on homologous parasite extract but also reacted as well with other potential surface-exposed antigens and/or cross-reacting ones. These findings are in good agreement with the results obtained by other studies (26, 34–35). On the other hand, mini-column cytoadherence technique was performed to identify and characterize the parasite surface antigens subsequently after selection. Results obtained here revealed that a high molecular weight protein in the TS fraction of the CHO, CHO/ ICAM-1 or CHO/CD36 binding subpopulations of A4 clone was identified which has been shown to be diverse. The protein was only detected in the protein extracts of selected subpopulations but not in uninfected erythrocytes or various CHO-cells. In addition, these molecules were recognized by the HIS, antibodies eluted from the surface of A4 clone and antibodies eluted from the intact cells bound to the ICAM-1 receptor. This protein was identified as having some of the characters of *Pf*EMP-1 e.g. the high molecular weight, surface location, trypsin sensitivity and parasite origin. The presence of *Pf*EMP-1 in the TS fraction of protein extract, which has previously shown to be insoluble in non-ionic detergents, could be attributed to the presence of the protein in the cytoplasm of parasite-infected cells. Previous studies have indicated that the Triton X-100-soluble pool represents intracellular *Pf*EMP-1, while the Triton X-100-insoluble pool represents *Pf*EMP-1 that has been delivered to knob complexes at the erythrocytes surface (5). But trypsin sensitivity, removing the protein by using 1 mg/ml trypsin, suggested that the protein was located on the surface of erythrocytes. It also suggested that the high expression of *Pf*EMP-1 must have occurred in the parasitized erythrocytes, which were able to bind to the endothelial receptors and that the interaction with receptors could change the character of this protein. Furthermore, the protein was not removed by 0.01 and 0.1 mg/ml of trypsin which showed that this molecule is resistant to low amounts of the enzyme (<1mg/ml). This is in good agreement with results of Chaiyaroj, colleagues (36) who indicated that PfEMP-1 of some *P. falciparum* isolates was trypsin resistant, and that they can remain cytoadherent after enzyme treatment. Moreover, Immunob-lot analysis of the TI fraction of the ICAM-1 binding subpopulation of *P. falciparum* A4 showed that at least three high molecular weight proteins were detected by both the HIS and the eluted antibodies from the surface of infected erythrocytes with A4. The range of molecular weight proteins was higher in ICAM-1 selected parasites compared to the parent population. Size increasing of *Pf*EMP-1 proteins in ICAM-1 binding subpopulation may be the result of amplification of a repetitive domain which is directly involved in cytoadherence. This amplification could lead to changes in other regions of the molecule, resulting in increase of cytoadherence as suggested by another study (37). This result was in good agreement with results obtained from other studies which indicated that the high molecular weight protein in adherent parasites is larger than unselected parasites (37–40). On the other hand, these results suggested that all three proteins could be *Pf*EMP-1 in view of the high molecular weight, insolubility in Triton X-100 and reaction with the eluted antibodies. In addition, the presence of more than one *Pf*EMP-1 in the ICAM-1 selected parasite may due to expression of different var gene which resulting in existence of mixed population with a similar character, binding to ICAM-1. Similarly, expression of several differently sized *Pf*EMP-1 bands in adhesion-selected clonal populations is shown in other experiments (40). More recently, a selected parasite sub-line expressed two different var genes as surface antigens on single infected erythrocytes, at the same time (41).

Another interesting band was ∼ 52-55 kDa in various *P. falciparum* isolates. This molecule as defined by its low molecular weight, Triton X-100 solubility, surface location and sensitivity to 1 mg/ml trypsin. The reaction of this band with eluted antibodies was stronger in comparison to HIS indicating considerable enrichment in the specificity of antibodies by affinity purification on the infected erythrocytes. These results suggested that the protein is parasite origin and may involve in cytoadherence because the band was only detected in the parasite protein extract and better detection in the ICAM-1 binding subpopulation of different isolate of *P. falciparum*. Additionally, in spite of non isolate-specific antibody response to this molecule it may be immunologically significant to the host immune system. These possibilities could be tested by inhibition of cytoadherence of infected erythrocytes and examining of antibody levels in hyper immune sera from malaria patients residing in different geographic regions. However, similar proteins have also shown by proteomics. These proteins are encoded by single copy genes and are highly conserved among *P. falciparum* isolates from various geographic locations (42).

## Conclusion

The surface antigens at infected erythrocytes membrane differed in parental population and various binding subpopulations of a particular isolate. These molecules could induce isolate-specific immunity. Furthermore, antibodies purified from the surface of infected erythrocytes can be used as specific reagents to investigate parasite derived proteins expressed on the surface of infected erythrocytes. These finding also reveled that the mini-column technique is a suitable method for preparing a reasonable amount of bound infected cells to a unique endothelial receptor. It made it possible to do further identification and characterization of the parasite antigens subsequently after selection.
